# 

**Published:** 1918-01-05

**Authors:** 


### CONTENTS.

| | PAGE |
|---|---|
| **A New Year Message** ... ... ... ... | 279 |
| **"That is Sound Which Works"** ... ... | 280 |
| **Hospital and Institutional News** ... ... | 281 |
| Hospital Development in the U.S.A.—A Medical Superintendent and a Secretary?—What is the Explanation?—The New Year Honours—Naval and Military Honours—Guardians and the Claims of the Hospitals—The Registration of Aliens—A Book of Appeals—War Savings in Scotland—Art and Sport Aiding Charity—The Latest *Gazette*—A Betrayal of Trust—Hun Professors in an Air-raid—The V.A.D. in Devonshire—This Week's Drug Market. | |
| **Glimpses Backward** ... ... ... ... ... ... | 285 |
| I.—Salerno and Medical Women. | |
| **A New Children's Hospital**... ... ... ... | 286 |
| **The Fashion to Decry Voluntaryism** ... ... | 287 |
| A Straight Talk on Hospital Finances: By the Right Hon. James W. Lowther, M.P., Speaker of the House of Commons. | |
| **Recently Published Parliamentary Returns, etc.** | 288 |
| **An Interlude and Two Reminiscences** ... ... | 289 |
| By "Caduceus." | |
| **The Modern Jew** ... ... ... ... ... | 290 |
| A Sociological and Biological Study. | |
| **The Institutional Library** ... ... ... ... | 291 |
| **The Matrons' and Sisters' Department** ... | 293 |
| Nursing Progress and its Developments: IV. Endowments for the College. | |
| Round the Hospitals. | |
| Forty-nine Christmas Days in Hospital—1917 Notable Over All—Personal Service Triumphant—The Bolos' Present Tactics—Reasons of their Failure—Trained Nurses and Parliament. | |
| **All Our Hospitals in Unity**... ... ... ... | 295 |
| The Best of the Old Year. | |
| **Our Hospitals' Christmas, 1917** ... ... ... | 298 |
| Bolo Buried and Forgotten Everywhere. | |
| St. Bartholomew's Hospital—St. Thomas's Hospital—At "The London"—Middlesex Hospital—St. Mary's Hospital—Metropolitan Hospital, Kingsland Road—Whipps Cross Infirmary and War Hospital—Brompton Hospital for Consumption—Bradford Royal Infirmary—The Leicester Hospitals—Norfolk and Norwich Hospital. | |
| **An Inquiry at Lambeth Infirmary** ... ... | 302 |
| Vindication of the Medical Staff. | |
| **The Institutional Worker** .. | (Suppl.) 1 |
| How to Organise a Mothers' and Babies' Welcome: Answer to November Question. | |

